# Patient Perspectives on Digital Technology and Experiences of Computerized History-Taking for Chest Pain Management in the Emergency Department: CLEOS-CPDS Prospective Cohort Study

**DOI:** 10.2196/65568

**Published:** 2025-06-17

**Authors:** Kaisa Fritzell, Helge Brandberg, Jonas Spaak, Sabine Koch, Carl Johan Sundberg, David Zakim, Thomas Kahan, Kay Sundberg

**Affiliations:** 1 Theme Cancer, Karolinska University Hospital, Karolinska vägen 27, Stockholm, 17176, Sweden, 46 761407424; 2 Division of Nursing, Department of Neurobiology, Care Sciences and Society, Karolinska Institutet, Alfred Nobels Allé 23, Huddinge, 14152, Sweden; 3 Division of Cardiovascular Medicine, Department of Clinical Sciences, Danderyd Hospital, Karolinska Institutet, Stockholm, Sweden; 4 Department of Learning, Informatics, Management and Ethics, Health Informatics Centre, Karolinska Institutet, Stockholm, Sweden; 5 Department of Learning, Informatics, Management and Ethics, Medical Management Centre, Karolinska Institutet, Stockholm, Sweden; 6 Department of Physiology & Pharmacology, Karolinska Institutet, Stockholm, Sweden

**Keywords:** technology acceptance, chest pain, computerized history taking, digital technology, emergency department, health informatics, medical history, self report

## Abstract

**Background:**

Automated, self-reported medical history-taking has the potential to provide comprehensive patient-reported data across a wide range of clinical issues. In the Clinical Expert Operating System‐Chest Pain Danderyd Study (CLEOS-CPDS), medical history data were entered by patients using tablets in an emergency department (ED). Since successful implementation of this technology depends on understanding patients’ views and willingness to use it, we have studied these factors following patients’ use of the CLEOS program.

**Objective:**

This study aimed to develop and use a questionnaire to investigate patients’ attitudes, perceptions and skills related to using digital technology in health care in general, and specifically their experiences with the CLEOS program during their visit to an ED with a chief complaint of chest pain.

**Methods:**

The study included the development of a questionnaire, followed by a cross-sectional study. Questionnaire design and the technology acceptance model underpinned the development of the questionnaire. The think-aloud method was used to test the questionnaire. Adults who participated in the CLEOS-CPDS were invited consecutively to respond to the questionnaire. Descriptive and correlational analyses were performed.

**Results:**

The refinement of the questionnaire included language revision, removal of similar items, and replacement of some response formats. The final questionnaire consisted of 16 items and one free text comment that assessed attitudes, perceptions, and skills related to the use of digital technology in health care in general and the specific experience of using self-reported history-taking by CLEOS. The majority of the 129 patients (mean age 56, SD=17.3 y) who answered the questionnaire found it easy to use digital technology in general (118/129, 91%), that digital technology has a role when seeking health care (115/129, 91%), and that patient-reported symptoms are helpful in making a diagnosis (83/129, 65%). There were some concerns that the patient-physician interaction would be disrupted when using digital technology (48/129, 38%). The overall experience of using CLEOS was positive and most felt confident in answering the questions on a tablet (118/129, 91%). Older age was associated with less ease (*P*<.001), confidence (*P*<.001), and trust (*P*=.002) when using digital technology, as well as less confidence in answering the questions in CLEOS (*P*=.019). Moreover, older age was associated with more worry about the potential disruption of the patient-physician personal contact when using digital technology (*P*<.001).

**Conclusions:**

This study suggests strong approval of usefulness and trust in digital technology among patients with chest pain visiting a cardiology ED, but the concern for lack of personal contact should be acknowledged. End users found the CLEOS program to perform well but recommend some adjustments for future studies. The questionnaire responses provided some new insights on perceived usability of digital technology for health care delivery, and it appears relevant for future evaluations of CLEOS in other contexts.

## Introduction

Automated, self-reported medical history-taking can become a key technology for maximizing outcomes for patients, for improving the physician-patient communication and for delivering patient-reported information to the point of care across a wide range of clinical issues [1,2]. Programs for computerized history-taking (CHT) that interact directly with patients in clinical settings collect more complete and accurate medical data as compared with physician entries in patients’ medical records [3,4]. For example, the CHT program Clinical Expert Operating System (CLEOS) provided sufficient data for risk stratification for a major adverse cardiac event using the well-established HEART (Heart, ECG, Age, Risk factors, Troponin) score in approximately 77% of 1000 emergency department (ED) patients with chest pain [5]. In contrast, risk stratification was only feasible for 31% of the patients when relying on data from the electronic health record [6]. CLEOS collects self-reported medical history data entered by patients on a tablet during hospital waiting times before or after seeing a physician [7]. The program emulates clinical thinking continuously through its automated interpretation of all previous answers as data is collected.

In recent years there has been a rapid growth of digital tools in health care that allow patients to interact with health care professionals [8]. Digital health tools can empower patients’ self-management and facilitate personalised communication with the health care system to improve health status awareness and increase adherence to therapy [8,9]. However, poor digital literacy, low health literacy, privacy and security concerns of medical data have been identified as barriers to adopting such tools [9]. As technical solutions are developed to improve health outcomes in patients with cardiovascular and other chronic conditions, patients’ ability to understand and communicate basic health information and to express their health concerns to health care professionals should be considered [10]. Furthermore, user-friendliness and facilitation of patient-clinician interaction have been reported as important aspects for the uptake and adoption of digital technology in health care [11]. Effective implementation of CHT programs in health care requires that we understand patients’ concerns, attitudes, and willingness to engage with such programs. Research on how CHT software is perceived by patients is very scarce. Early work in this field suggests that CHT is well accepted [12,13] but older age and low self-confidence in using computers were associated with lower perceived usability [13]. In a more recent study of the usability of an application designed for medical history-taking in general practice the participants of older age did not adapt equally well to the application [14]. We conclude that the ongoing development in technology and changes in health care with people seeking care at increasingly older ages requires an extended knowledge of patients’ views on these issues.

We previously performed an interview study of patients seeking ED care for chest pain as part of our ongoing studies of patient experience with the use of CLEOS [15]. The interviews yielded insight into the influence of context, clinical content, and technology on the program’s usability. Generally, CLEOS was well accepted by the patients and could be managed by them despite a busy ED, although the program was sometimes perceived too extensive. However, the sample interviewed was small and we did not address patients’ perceptions of ease of using the program and its possible impact on the value of health care they could receive. These are 2 determinants identified in the technology acceptance model (TAM) for acceptance of computer technology by its potential users [16]. Obviously, effective development of patient-directed health care technology depends on thorough understanding of patient perceptions of the technology.

Most previous studies focus on digital health care tools aimed at delivering interventions, patient monitoring, symptom tracking, and communication. A study of patient perceptions of CHT based on real-time use by patients with significant medical issues has not been investigated in detail. This study will provide insights for developers of CHT and health care managers on important factors that influence an individual’s intention to use such programs effectively as they seek emergency health care. Accordingly, this study aimed to develop a questionnaire and apply it to investigate patients’ attitudes, perceptions, and skills related to using digital technology in health care in general, and specifically their experiences with the CLEOS program in an ED setting.

## Methods

### Design

The study design included (1) the development of a questionnaire and (2) a cross-sectional study using the questionnaire in a study population presenting to the ED with acute chest pain. This study is part of the Clinical Expert Operating System ‐ Chest Pain Danderyd Study (CLEOS‐CPDS) presented in detail elsewhere [7].

### Questionnaire Development

#### Development

The questionnaire development was guided by questionnaire design [17,18] and by results from our previous interview study investigating patients interacting with CLEOS in an ED [15]. The construction of items in the questionnaire was inspired by the System Usability Scale (SUS) [19]. The SUS is a standardized questionnaire for the assessment of perceived usability. It does not cover all areas of significance for the research questions we address here. Therefore, we used the TAM [16] to give further structure to the development of questions. The TAM determines an individual’s evaluative judgment of the target behavior in some dimension (attitudes). For example, it includes the perception that using an IT system will be free of effort (perceived ease of use) and will enhance performance (perceived usefulness). Another determinant in the TAM is the specific behavior when interacting with the technology of the CHT program (use). The Swedish functional health literacy scale [20] was used for inspiration to develop questions to evaluate an individual’s digital health literacy (skills). All areas of interest (attitudes, perceptions, use and skills) were entered into a template guiding the construction of the questionnaire (Multimedia Appendix 1). Furthermore, 2 scaling methods were chosen initially for capturing patients’ responses in the questionnaire. These were the Likert method (strongly agree, agree, undecided, disagree, and strongly disagree), and a frequency scale with fixed choice responses (always, often, sometimes, seldom, and never).

#### Face Validity Test of the Questionnaire

In January 2022, 14 patients visiting the cardiology ED at Danderyd University Hospital with acute chest pain and taking part in the CLEOS-CPDS prospective cohort study were asked to participate in the development of the questionnaire. The selected patients represented a variation in age and gender. If they agreed to participate, an information letter and a questionnaire were sent to their home by post; and a few days later a researcher (KS or KF) contacted them by phone. We used the think-aloud (TA) method [21] to test the face validity of the questionnaire. During the phone call, the respondents were asked to fill in the questionnaire, encouraged to think out loud and to verbalize any thoughts while doing so. This provided an opportunity to listen to their understanding of each item as they worked through the questionnaire. In addition, probing questions were asked when the session leader needed further clarification. At the end of the TA session, all participants were asked if they had any additional comments that had not been addressed during the session. The content of the sessions was recorded in a spreadsheet to facilitate the evaluation and revision of the questionnaire.

### Investigating Outcomes of the Questionnaire

#### Sample and Setting

Adult patients (≥18 y) with acute chest pain, presenting to the ED at Danderyd University Hospital and participating in the CLEOS-CPDS from May until November 2022 (n=288, mean age 55 y, 41% women) were invited to answer the questionnaire. Before discharge from the ED, patients were invited consecutively by a study nurse to participate. Those who agreed were given study information and a paper copy of the questionnaire, along with a prepaid return envelope. Of the 130 questionnaires returned, one was blank, leaving 129 participants. The mean age of the patients participating was 56 (SD 17.3) years (range 18-89 y). More men (79/129, 61%) than women (49/129, 38%) participated (Table 1).

**Table 1.** Sociodemographic characteristics for participants answering the questionnaire (N=129).

Characteristics	
Age (years), mean (minimum-maximum)	56 (18-89)
Gender [1], n (%)	
Women	49 (38)
Men	79 (61)
Missing	1 (<1)
Education level, n (%)	
Junior compulsory	7 (5)
Senior high school	47 (37)
Postgraduate or university	74 (58)
Missing	1 (<1)
Occupation, n (%)	
Working or student	87 (67)
Retired	40 (31)
Unemployed or sick leave	2 (2)

### Analysis

The items in the questionnaire were analyzed using descriptive statistics, including frequencies and percentages. Spearman’s rho was used to explore the correlations between age, attitudes of using digital technology and experience of using CLEOS. For analysis in SPSS Statistics 30 (IBM), the responses were labeled as “always”=1, “often”=2, “sometimes”=3, “seldom”=4, or “never”=5. The responses were ranked from low to high, with the item “always” given the lowest ranking of 5 and the item “never” the highest rank of 1. Thus, a positive correlation means a relationship between older age and “never” and a negative correlation means a strong relationship between older age and “always.”

Internal item consistency was tested for 6 items corresponding to questions on health literacy: “I could make use of the content in the text, “It took a long time for me to read the text”, “There were words that I did not understand,” “It was difficult to answer the questions,” “The questions were relevant,” “It was easy to find relevant response alternatives.” The intended health literacy scale had a Cronbach α of .68. The items corresponding to attitudes, perceptions and skills were too diverse to make up a scale and were hence not tested for internal consistency.

Conventional content analysis was applied for the free text comments [22]. The analysis was conducted by 2 of the authors (KF and KS), both experienced with qualitative research. The comments were entered into a document and coded by highlighting similar texts in the same color, followed by categorizing them into color-coded clusters. The clusters were defined into categories which were discussed and refined and then finalized in agreement with all authors. A response frequency chart was used to determine the frequency of similar comments.

### Ethical Considerations

This study has been approved by the Swedish Ethical Review Authority (reference number 2015/1955‐31) and is registered at ClinicalTrials.gov, National Library of Medicine, (NCT03439449). Informed consent was not obtained specifically for this study since patients had been informed. while obtainging informed consent in the CLEOS-CPDS study, that they might be asked later to take part in an evaluation of their perceptions of using the programme. To ensure that no participants could be identified, all participants names were removed and replaced with an unique code. No compensations for participating in this study were offered.

## Results

### Development of the Questionnaire

The initial version of the questionnaire contained a total of 19 items and an additional 4 sociodemographic questions. The items concerned experience of use of digital technology in general, the use of digital technology in health care, and the use of the CLEOS program.

In addition, 12 individual TA sessions were held, involving 6 women and 6 men, with mean age 72 (range 37-82) years. A majority had postgraduate or university (n=7) as the highest level of education, followed by senior high school (n=2), junior compulsory (n=3), and a majority were retired (n=8).

Based on the results of the TA sessions, the wording of 2 questionnaire items was revised. For item 5, the word “lean on” was changed to “depend on” and for item 7, the word “relation” was changed to “personal contact.” Three items were removed because they resembled other items and did not contribute to the evaluation of CLEOS. Moreover, the response format “strongly agree” to “strongly disagree,” which was used for seven items, was perceived as difficult to understand. It was replaced with the response format “always,” “often,” “sometimes,” “seldom” or “never,” which was consistent with the allowed response alternatives for all other items. The revised questionnaire had 16 items and 1 free text comment (Multimedia Appendix 2). The first set of questions (n=9) assessed attitudes, perceptions, and skills related to the use of digital technology in health care (Tables2  3). The second set (n=7) evaluated the specific experience of self-reported history-taking by interacting with CLEOS (Table 4). The questionnaire included a combination of positively and negatively phrased items, response alternatives such as (yes, no, uncertain), and frequency scales (always, often, sometimes, seldom, never).

**Table 2.** Attitudes, perceptions, and skills related to the use of digital technology in health care (N=127).

	Yes, n (%)	No, n (%)	Uncertain, n (%)
Have encountered digital technology previously in contact with health care	63 (50)	59 (46)	5 (4)
Digital technology has a role when visiting health care	115 (91)	2 (1)	10 (8)

**Table 3.** Summary of attitudes, perceptions, and skills toward digital technology use in health care.

	Always, n (%)	Often, n (%)	Sometimes, n (%)	Seldom, n (%)	Never, n (%)	n
Find it easy using digital technology	104 (80)	14 (11)	9 (7)	1 (1)	1 (1)	129
Feel confident using digital technology	105 (81)	13 (10)	7 (5)	3 (2)	1 (1)	129
Trust digital technology to function as intended when visiting health care	84 (66)	31 (24)	10 (8)	0	2 (2)	127
Worry that information regarding own health collected by digital technology will be disclosed to unauthorized persons	4 (3)	1 (1)	18 (14)	31 (24)	73 (58)	127
Worry that the patient-doctor personal contact is disturbed when digital technology is used in health care	11 (9)	3 (2)	34 (27)	22 (17)	58 (45)	128
Believe that patient-reported symptoms using digital technology are helpful for the physician when making a diagnosis	60 (47)	23 (18)	35 (28)	6 (5)	3 (2)	127
Believe that patient contribution is valuable when developing digital technology in health care	100 (78)	17 (!4)	8 (6)	3 (2)	0	128

**Table 4.** Experience of answering questions regarding own health using Clinical Expert Operating System on a tablet.

Experiences	Always, n (%)	Often, n (%)	Sometimes, n (%)	Seldom, n (%)	Never, n (%)	n
I was confident answering the questions	90 (70)	25 (20)	11 (9)	1 (1)	1 (1)	128
I could make use of the content in the text	92 (71)	33 (26)	3 (2)	0	1 (1)	129
It took a long time for me to read the text	5 (4)	5 (4)	23 (18)	21 (16)	75 (58)	129
There were words that I did not understand	6 (5)	1 (<1)	11 (9)	26 (20)	85 (66)	129
It was difficult to answer the questions	2 (2)	4 (3)	34 (26)	36 (28)	53 (41)	129
The questions were relevant	46 (36)	47 (36)	32 (25)	4 (3)	0	129
It was easy to find relevant response alternatives	34 (26)	68 (53)	26 (20)	0	1 (1)	129

### Outcomes of the Questionnaire

#### Attitudes, Perceptions, and Skills

Most participants found it easy or often easy (n=118, 91%) and felt confident or often confident (n=118, 91%) using digital technology. Most (n=115, 91%) believed that digital technology has a role when visiting health care. Half (n=63, 50%) had not previously encountered or were not sure if they had encountered digital technology in interactions with healthcare. Most (n=115, 90%) trusted digital technology to function as intended during health care visits. One-fifth (n=23, 18%) was concerned or sometimes concerned that information regarding their health collected by digital technology would be disclosed to unauthorized persons. More than one-third (n=48, 38%) was concerned or sometimes concerned that the interaction between patient and doctor would be disrupted when using digital technology in health care. In addition, two-thirds (N=83, 65%) believed that patient reported symptoms using digital technology are helpful for the doctor in making a diagnosis. Four-fifths (n=100, 78%) believed that patient contribution is valuable when developing digital technology in health care (Table 2 and 3).

#### Experience of Using CLEOS

Most respondents (n=115, 90%) were confident or often confident to answer the questions on a tablet in the CLEOS-CPDS study. Almost all (n=125, 97%) could make use of the content in the text. Most (n=111, 86%) reported a high level of understanding of the terminology used in the CLEOS program, and a majority (n=89, 69%) did not or seldom find that the questions were difficult to answer. A majority of respondents (n=93, 72%) found that the questions in the CLEOS program were relevant. Slightly more (n=102, 79%) thought it had been easy or often easy to find relevant response alternatives (Table 4).

#### Correlations Between Age and Attitudes, and the Experience of Using Digital Technology

Significant, moderately positive correlations were found between age and the items “I find it easy using digital technology” (*P*=<.001; 95% CI 0.171-0.492), “I feel confident using digital technology in general” (*P*<.001; 95% CI 0.247-0.551), “I trust the digital technology to function as intended when visiting healthcare“ (*P*=.002) and “I was confident answering the questions in Cleos” (*P*=.02, 95% CI 0.032-0.379). The items were rated as “always,” “often,” “sometimes,” “seldom,” and “never” whereas “always” had the lowest ranking and “never” the highest ranking. The correlations indicate that older age is associated with less ease, confidence, and trust in using digital technology as well as confidence in answering the questions. A significant negative correlation was found between “I worry that the patient-doctor personal contact is disturbed when digital technology is used in healthcare” (*P*=.001; 95% CI −.443 to −.107), rated as “always,” “often,” “sometimes,” “seldom,” and “never,” indicating that higher age is associated with greater concern (Table 5). The results for 127‐129 participants were tested using Spearman rho. The ranking was from low to high; the item “always” has the lowest ranking of 5, and the item “never” has the highest rank of 1. A significant positive correlation means a relationship between older age and “never” and a significant negative correlation means a strong relationship between older age and “always.”

**Table 5.** Correlations between age and attitudes related to the use of digital technology in health care and experience of answering questions using clinical expert operating system (N=129).

	Correlation coefficient	*P* value	95% CI
Attitudes of using digital technology^a^			
Find it easy using digital technology	0.342	<. 001	.171 to .492
Feel confident using digital technology	0.41	<. 001	.247 to .551
Trust digital technology to function as intended when visiting health care	0.278	.002	.100 to .439
Worry that information regarding own health collected by digital technology will be disclosed to unauthorized persons	–0.078	.39	–.257 to –.107
Worry that the patient-doctor personal contact is disrupted when digital technology is used in health care	–0.283	.001	–.443 to –.107
Believe patient-reported symptoms using digital technology are helpful for the physician when making a diagnosis	–0.02	.83	–.202 to .163
Believe that patient contribution is valuable when developing digital technology in health care	0.129	.15	–.054 to .304
Experience of answering questions			
I was confident answering the questions	0.212	.02	.031 to .379
I could make use of the content in the text	0.035	.70	–.148 to .215
It took a long time for me to read the text	–0.032	.72	–.213 to .150
There were words that I did not understand	0.097	0.28	–.086 to .274
It was difficult to answer the questions	–0.16	.08	–.332 to .022
The questions were relevant	–0.161	.07	–.333 to .021
It was easy to find relevant response alternatives	0.04	.662	–.142 to .220

a Results for 127‐129 participants. Tested by Spearman´s rho. Ranking (from low to high) the item “always” has the lowest ranking of 5, and the item “never” has the highest rank of 1. A significant positive correlation means a relationship between older age and “never” and a significant negative correlation means a strong relationship between older age and “always.”

#### Free Text Comments

The last item in the questionnaire allowed patients to comment on their experience of performing the CLEOS interview on the tablet (Textbox 1). A total of 40% of the patients left comments concerning (1) time aspects regarding the CLEOS interview (n=11), perceptions regarding the questions included in CLEOS (n=13), (2) perceptions regarding the response alternatives (n=13), (3) perceptions of technical performance (n=6), (4) perceptions of layout (n=9), (5) difficult-to-understand words (n=6), and (6) not confident (n=2).

Textbox 1. **Categories and examples of comments in the questionnaire regarding answering questions in CLEOS.**

**Time aspects regarding the clinical expert operating system interview (n=11)**

It was too extensive and time consuming, it was tiresome to answer all the questions and hard to stay focused, not enough time to answer all questions.

**Perceptions regarding the questions (n=13**)

Lacking questions (ie, a question about other diseases), questions that were grammatically wrong, not translated from German or English, questions about a topic that never ended or unrecognizable scenarios becoming increasingly irrelevant, questions that were incomplete and lacking an illustration (image), irrelevant follow-up question (ie, requested to answer “what kind of surgery” after having said no to “had any surgery”).

**Perceptions regarding the response alternatives (n=13**)

Lacking an appropriate alternative, wanting more alternatives or “I don’t know”, having to answer “how often” after answering no to a symptom occurrence, not finding a prescribed medication in the list of medicines.

**Perceptions of technical performance (n=6**)

A slow interface, technical, or internet problems.

**Perceptions of layout (n=9**)

The size of the text was too small, unclear transition to the next page, not clear when the questions had ended, an inert maneuvering.

**Difficult to understand words (n=6**)

Connotation of wording that was, that some words needed explanation, that medical terms were difficult, some text was strangely worded.

**Not confident (n=2**)

Feeling uncertain about using the technique, “talking” to a doctor on a screen feels impersonal.

## Discussion

### Principal Findings

This study included a process of developing a questionnaire and a cross-sectional study that evaluated patients’ attitudes toward digital technology in health care and experiences of interacting with a CHT program while presenting to an ED setting with acute chest pain. The questionnaire development process required only minor changes for the final version. Overall, the findings from the evaluation of using the CLEOS program showed a high acceptance of digital technology when incorporated in health care as well as user confidence and high digital literacy among the participants.

### Comparison With Previous Work

In general, there was a favorable attitude toward digital technology, with many participants expressing their faith in its potential to help physicians make accurate diagnoses. Although not all had previous experience using digital technology in health care encounters, a clear majority in this study had a positive attitude about the role of such technology when visiting health care. Moreover, most were confident using the CLEOS program. This is in line with the results of Arora et al [12] who were early to use a CHT program in the waiting area of an ED. In that study, the overall impression among the patients was that they would like to fill out a digital questionnaire again in the future. In a more recent pilot study assessing digital patient self-anamnesis in the waiting area of an ED, 63% agreed to fill out a digital questionnaire again [23]. In another study evaluating medical history-taking via an app in general practice, the usability score (evaluated by SUS) was in favor of acknowledging a recurrent use of the application [14]. The application described in that study is similar to a CHT program that emulates clinical thinking, but the application uses a branching logic that is adaptive to patient responses instead of automated interpretation of previous answers. While a clear majority expressed a trust in the technique when used in health care interactions, there were some concerns about data security and privacy in the present study. The high level of trust in digital technology to function well was somewhat surprising in our present results, given that only just over half of the respondents had previously encountered digital technology when in contact with health care. Altogether, the results from our current study support previous findings of a high level of patient acceptance of health care technology when visiting EDs.

There was a modest but noteworthy concern about disrupted patient-physician interaction when using digital technology in health care. This concern was more pronounced in older patients. As there are no other CHT programs like CLEOS and very few other comparable systems, it is hard to conclude what these results mean. A related area in health care, however, is patients’ opinions about the patient-physician relationship and communication with physicians using electronic medical records (EMR). Perceptions of such computer use during the health care visit were compiled in a systematic review. The authors concluded that it did not substantially affect the quality of communication with their physician [24]. In a survey examining perceived benefits and risks of using artificial intelligence (AI) applications in health care, communication barriers emerged as the most significant predictors of perceived risks [25]. The authors discussed that the loss of face-to-face cues and lack of interaction with physicians would give the patient a more passive position. On the other hand, their results showed that if users believe that AI-based devices can improve diagnostics and patient management systems, they become more prone to use them. A review showed that digital tools in health care can be facilitators for factors such as empowerment and personalized communication with health care [9]. Reciprocally, patient empowerment contributed to patient uptake of digital health tools. The study concluded too that digital tools could facilitate a shift from paternalistic health care models to those in which relationships between clinicians and patients are more collaborative. Based on these findings, we agree that patient-physician communication through modern technology could be perceived as positive by patients. Nevertheless, our results also tell us that patients’ trust, especially with rising age, needs to be ensured and further investigated before CHT programs are integrated into standard clinical practice.

The ability to understand and make use of the content in the CLEOS program was overall very satisfactory. Low digital and health literacy have been described as barriers to implementation of digital tools [9,10]. Our results may be a consequence of a presumed high health literacy among the participants related to a relatively high educational level, known as a strong associated factor [26]. Higher age could be another barrier. The present results suggest that older people are more likely to experience less ease and confidence in using digital technology. This could explain why being 70 years or older was found to be a negative factor for completing the program in a previous study of the CLEOS-CPDS cohort [5]. Similarly, Albrink et al [14] found that age was negatively associated with the usability SUS score when evaluating their app, suggesting that older people may face challenges in handling such an app.

Our study shows strong support for involving end users when designing new digital technology in health care. This confirms findings by others who recommend a participatory approach in the design of future digital health care [27-29]. The free text comments revealed aspects for improvement of the CLEOS program such as high time consumption, ambiguous questions, and slow technical performance. These results align well with those from our previous interview study [15], highlighting the need for further development of the program.

The challenge of developing questionnaires in research is to achieve high validity and reliability. Accordingly, the questionnaire development was guided by a robust process based on the results of our previous interview study and other recognized methods. Furthermore, the development process included the think-aloud method [21], which allowed the participants to speak freely while testing the questionnaire. The think-aloud method has no specific sample size recommendation, and we decided to stop inclusion when no new data was collected. Although the sample in the present study may be small, they provided valuable information about the comprehensibility of the questionnaire. Presumably, all areas of interest were covered sufficiently. However, to be sure that the questionnaire measures what is intended, a more robust validation in a future study is needed.

Our results should be interpreted in the context of the study’s limitations. The convenience sample of patients responding to the questionnaire may not be representative of all patients seeking care at the cardiology ED. The small sample size and single center inclusion affects the generalizability of the results. Further, the selected sample had been consecutively recruited to the CLEOS-CPDS study and may have had a positive attitude toward digital technology as they had consented to participate in the study. Other selection biases could be a slight overrepresentation of men in the sample and an uneven distribution of education levels. Furthermore, we lack information about those who did not fulfil the criteria for inclusion, eg, fluency in Swedish, or declined participation both in CLEOS-CPDS and in the current study.

### Conclusion

This study suggests strong acceptability and trust of digital technology among patients presenting to a cardiology ED. However, the concern for lack of personal contact between patient and doctor should be acknowledged. Digital technology was seen as an important facilitator but not as a replacement for human interaction. The CHT program (CLEOS) performed well for end-users but would benefit from some adjustments for future studies. The questionnaire responses yielded some new insights into the use of digital technology for health care delivery. The questionnaire appears relevant and may be useful for future evaluations of CHT in various contexts.

## Supplementary material

10.2196/65568 Multimedia Appendix 1 Template of the development of the questionnaire.

10.2196/65568 Multimedia Appendix 2 The questionnaire.

## Abbreviations

AI: artificial intelligence
CHT: computerized history-taking
CLEOS: clinical expert operating system
CLEOS-CPDS: Clinical Expert Operating System - chest pain Danderyd study
ED: emergency department
EMR: electronic medical records
HEART: history, electrocardiogram, age, risk factors and troponin
SUS: System Usability Scale
TA: think-aloud
TAM: Technology Acceptance Model
