# Supplementary material for: Patient Perspectives on Digital Technology and Experiences of Computerized History-Taking for Chest Pain Management in the Emergency Department: CLEOS-CPDS Prospective Cohort Study
Source: JMIR Med Inform. 2025 Jun 17;13:e65568. doi: 10.2196/65568 (PMC12187027; doi:10.2196/65568)
Supplement: Multimedia Appendix 1 [file medinform-v13-e65568-s001.pdf]

Appendix 1: Template for developing the questionnaire inspired by the Technology Acceptance Model

**Purpose:** To investigate the patient's attitudes, perceptions, use and skills when using digital technology in healthcare and the computerized history taking program (CHT), CLEOS				
**Areas of interest**	**Attitudes**	**Perceptions**	**Use**	**Skills**
**Attitude (ATT)**<br>An individual's evaluative judgment of the target behavior on some dimension (e.g., good/bad, harmful/beneficial, pleasant/unpleasant)<br>**Perceived ease of use (PEOU)**<br>An individual's perception that using an IT system will be free of effort<br>**Perceived usefulness (PU)**<br>An individual's perception that using an IT system will enhance job performance<br>**Use (USE)**<br>One specific behavior of interest performed by individuals regarding some information technology (IT) system<br>**Skills**<br>Digital literacy, health literacy	• Degree of beliefs that an IT system (in general) exhibit technological uncertainties.<br>• The extent of perceived privacy concerns regarding personal information.<br>• Ranking the trustworthiness of type of organization that collects health data<br>• Degree to feelings that an CHT has an impact on relations to physician.<br>• The extent of concerns regarding data storage.<br>• Degree of beliefs that CHT delivers trustworthy information.<br>• The extent of perceived risks to use CHT (quality of diagnosis).<br>• The extent of perceived benefits to use CHT-program (improve diagnostics).	• Degree of comfort of using CHT in ED.<br>• Degree of comfort using CHT regarding health condition.<br>• Degree of belief that patient's role (reporting medical history) is important in the diagnostic process.<br>• Importance of patient contribution in development phase.	• General impression of using CHT.<br>• The extent of perceived relevance of the CHT content.<br>• The extent of user-friendliness of CHT.	• General rating of digital/computer skills<br>• Having used any similar program in health care<br>• Rating of confidence in using CHT.<br>• Rating of health literacy skills (inspired by the 5-item HL-scale)
