# Supplementary material for: Patient Perspectives on Digital Technology and Experiences of Computerized History-Taking for Chest Pain Management in the Emergency Department: CLEOS-CPDS Prospective Cohort Study
Source: JMIR Med Inform. 2025 Jun 17;13:e65568. doi: 10.2196/65568 (PMC12187027; doi:10.2196/65568)
Supplement: Multimedia Appendix 2 [file medinform-v13-e65568-s002.pdf]

Appendix 2. CLEOS perception scale

When you visited the emergency department for chest pain you were asked to participate in a study where you answered questions regarding your health with the support of digital technology (on a tablet). To evaluate your experience, we ask you to answer the questions below.

The questions involve previous experiences of digital technology (i.e. computer, tablet, smartphone) and attitudes to digital technology in health care.

The following questions concern using digital technology in general

1. I find it easy using digital technology (always, often, sometimes, seldom, never)
2. I feel confident using digital technology (always, often, sometimes, seldom, never)

Following questions concern using digital technology when utilizing healthcare

3. Have you encountered digital technology previously in your contact with health care? (yes, no, uncertain)
4. Do you think that digital technology has a role when visiting health care? (yes, no, uncertain)
5. I trust the digital technology to function as intended when visiting health care (always, often, sometimes, seldom, never)
6. I worry that information regarding my health collected by digital technology will be disclosed to unauthorized persons (always, often, sometimes, seldom, never)
7. I worry that the patient doctor personal contact is disturbed when digital technology is used in health care (always, often, sometimes, seldom, never)
8. I think that patient reported symptoms using digital technology are helpful for the physician when making a diagnosis (always, often, sometimes, seldom, never)
9. I think that patient contribution is valuable when developing digital technology in health care (always, often, sometimes, seldom, never)

The following questions concerns your experience of answering questions regarding your health using a tablet in the CLEOS study.

10. I was confident answering the questions (always, often, sometimes, seldom, never)
11. I could utilize the content in the text (always, often, sometimes, seldom, never)
12. It took a long time for me to read the text (always, often, sometimes, seldom, never)
13. There were words that I did not understand (always, often, sometimes, seldom, never)
14. The questions were relevant (always, often, sometimes, seldom, never)
15. It was easy to find relevant response alternatives (always, often, sometimes, seldom, never)
16. It was difficult to answer the questions (always, often, sometimes, seldom, never)
17. Do you have any other comments regarding your experience of answering questions in CLEOS (free text)?
